# Sufficient Thrombin Generation Despite 95% Hemodilution: An In Vitro Experimental Study

**DOI:** 10.3390/jcm9123805

**Published:** 2020-11-25

**Authors:** Johannes Gratz, Christoph J. Schlimp, Markus Honickel, Nadine Hochhausen, Herbert Schöchl, Oliver Grottke

**Affiliations:** 1 Department of Anaesthesiology, Intensive Care Medicine and Pain Medicine, Medical University of Vienna, Waehringer Guertel 18–20, 1090 Vienna, Austria; johannes.gratz@meduniwien.ac.at; 2 Ludwig Boltzmann Institute for Experimental and Clinical Traumatology, AUVA Research Centre, Donaueschingenstraße 13, 1200 Vienna, Austria; christoph.schlimp@trauma.lbg.ac.at (C.J.S.); herbert.schoechl@auva.at (H.S.); 3 Department of Anesthesiology and Intensive Care Medicine, AUVA Trauma Centre Linz, Garnisonstrasse 7, 4010 Linz, Austria; 4 Department of Anesthesiology, University Hospital of the RWTH Aachen, Pauwelsstrasse 30, 52074 Aachen, Germany; mhonickel@ukaachen.de (M.H.); nhochhausen@ukaachen.de (N.H.); 5 Department of Anaesthesia and Intensive Care Medicine, AUVA Trauma Centre Salzburg, Academic Teaching Hospital of the Paracelsus Medical University Salzburg, Doktor-Franz-Rehrl-Platz 5, 5010 Salzburg, Austria

**Keywords:** coagulation, factor concentrates, hemorrhage, massive transfusion

## Abstract

Guidelines for the treatment of severe bleeding comprise viscoelastic-test-guided use of coagulation factor concentrates as part of their recommendations. The aim of this study is to investigate the effects of substituting fibrinogen, prothrombin complex concentrate, and a combination of both on conventional coagulation tests, viscoelastic test results, and thrombin generation. Blood was drawn from seven healthy volunteers to obtain platelet-free plasma, which later was diluted by replacing 40%, 60%, 80%, 90%, 95%, and 99% with a crystalloid solution. The diluted samples were spiked with fibrinogen concentrate, prothrombin complex concentrate, a combination of both, or a corresponding amount of crystalloid solution. Up to a dilution level of 95%, viscoelastically determined clotting time was significantly shorter in the group substituted with fibrinogen only in comparison with the additional use of prothrombin complex concentrate. Clot firmness and endogenous thrombin potential remained at relatively stable values up to a dilution level of 95% with the substitution of fibrinogen but not prothrombin complex concentrate. Substitution of prothrombin complex concentrate led to an excessive overshoot of thrombin generation. The results of our study question currently propagated treatment algorithms for bleeding patients that include the use of prothrombin complex concentrate for patients without former intake of oral anticoagulants. Even in severely bleeding patients, thrombin generation might be sufficient to achieve adequate hemostasis.

## 1. Introduction

Hemorrhage in severely injured patients is associated with high mortality and is the leading cause of death in trauma patients [1]. One-third of bleeding trauma patients exhibit signs of trauma-induced coagulopathy, which is directly associated with an impaired outcome [2]. Historically, explanatory models for trauma-induced coagulopathy have focused on blood loss, dilution of coagulation factors due to volume replacement, hypothermia, and acidosis [3]. Although these factors might play a role in the development of trauma-induced coagulopathy, recent literature provides a more differentiated picture, characterizing it as a multifaceted process, including many more drivers such as shock-related hypoperfusion, endothelial injury with glycocalyx shedding, and platelet dysfunction [4,5].

Alongside rapid bleeding control, restrictive volume replacement, and the early use of tranexamic acid, current trauma guidelines comprise the administration of coagulation factor concentrates such as fibrinogen and prothrombin complex concentrate as part of their recommendations [6]. Fibrinogen is the first single coagulation factor that drops beyond critical levels in bleeding patients [7], and low fibrinogen levels upon hospital admission have been associated with increased mortality in trauma patients [8]. Thus, maintaining plasma fibrinogen levels ≥1.5 g/L is one of the cornerstones in hemostatic management of bleeding trauma patients [6].

While many centers worldwide use a fixed-ratio approach of packed red blood cells, platelet concentrates, and fresh frozen plasma for the management of severely injured trauma patients, a number of European trauma centers have implemented goal-directed treatment algorithms [9,10,11,12,13]. These primarily rely on prompt administration of tranexamic acid and the early use of fibrinogen concentrate as first-line therapy. Subsequently, prothrombin complex concentrate is administered according to point-of-care-available viscoelastic test results. Due to a number of advantages over conventional coagulation tests, viscoelastic testing is increasingly used to guide hemostatic therapy in bleeding trauma patients [14,15,16,17]. Several treatment algorithms have been suggested that, among other parameters, rely on the viscoelastic measurement of the initiation phase of the coagulation process, such as r-time (thrombelastography) or clotting time (rotational thromboelastometry), to trigger administration of prothrombin complex concentrate. However, to what extent these parameters are influenced by thrombin generation or substrates such as fibrinogen or platelets remains to be elucidated. Importantly, the use of prothrombin complex concentrate in bleeding patients does not come without significant side effects and has been associated with rates of up to 41% of thromboembolic events [18].

In an experimental model of extreme hemodilution, we thus investigated the effects of substituting (1) fibrinogen, (2) prothrombin complex concentrate, and (3) a combination of both on conventional coagulation tests, viscoelastic test results, and thrombin generation.

## 2. Experimental Section

The present study was approved by the relevant local ethics committee (RWTH Aachen, EK 380/15). Figure S1 gives an overview of the experimental procedures. After obtaining written informed consent, blood was drawn into citrated plasma tubes (Sarstedt, Nümbrecht, Germany) from a total of seven healthy volunteers by antecubital venipuncture. All participants did not have any history of coagulopathy, thromboembolism, or intake of antiplatelet or anticoagulant medication at the time of inclusion.

The blood samples were double-centrifuged to obtain platelet-free plasma. Thereafter, platelet-free plasma was diluted by replacing 40%, 60%, 80%, 90%, 95%, and 99% with a balanced crystalloid solution (Sterofundin, B.Braun, Melsungen, Germany). The resulting diluted samples were spiked with (1) FC group: fibrinogen concentrate (FC; Haemocomplettan P, CSL Behring, Marburg, Germany); (2) PCC group: four-factor prothrombin complex concentrate (PCC; Beriplex P/N, CSL Behring, Marburg, Germany); (3) FC+PCC group: a combination of FC+PCC; (4) control group: a corresponding amount of balanced crystalloid solution. The diluted and spiked plasma samples of the four study groups were stored at −80 °C until the commencement of analysis.

For the preparation of the fibrinogen groups (FC, FC+PCC), the baseline individual plasma fibrinogen concentrations were measured and set as the target fibrinogen levels for substitution. Expected fibrinogen levels were calculated for the different dilutions, and fibrinogen concentrate was added accordingly. For the preparation of the PCC groups (PCC, FC+PCC), PCC was substituted in order to keep the prothrombin time index (PTI) at approximately 100%. PTI was expected to decrease in correlation to dilution levels. Thus, for example, at the 40% dilution level, PCC was added to raise PTI by 40% according to the prescribing information.

The resulting samples were then characterized by a number of measurements. Fibrinogen concentration was assessed on a coagulometer (ACL-TOP 550, Werfen, Munich, Germany). Prothrombin time, activated partial thromboplastin time, factor II levels, and antithrombin activity were determined with appropriate reagents on a hematology analyzer (ACL TOP 550; Werfen, Munich, Germany). Thromboelastometry (ROTEM; Tem International, Munich, Germany) was used for the measurement of viscoelastic coagulation properties of the different samples. The extrinsically activated assay using recombinant tissue factor (EXTEM) was performed, and clotting time (CT) and maximum clot firmness (MCF) were analyzed. Furthermore, for measurement of thrombin generation, we used the calibrated automated thrombogram method, as previously described [19], calculating thrombin generation curves with Thrombinoscope software (Thrombinoscope BV; Maastricht, The Netherlands).

Because of thrombin generation measurements beyond the upper detectable limit at higher dilution rates in the two PCC groups (PCC, FC+PCC), we decided to substitute antithrombin in those groups and repeat the measurements. Thus, in a second analysis step, samples in the PCC groups were spiked with antithrombin (Kybernin P, CSL Behring, Marburg, Germany) in order to keep antithrombin activity at approximately 100%, and thrombin generation measurements were repeated, as described above.

Since this study was designed as a pilot, no sample size calculation was performed. Data distribution was assessed by visual inspection and the use of the Kolmogorov–Smirnov test. According to normal data distribution results were expressed as mean (SD). One-way ANOVA and Tukey’s posthoc comparison were used to test for differences between the study groups. A two-sided *p*-value <0.05 was considered statistically significant. Statistical analysis was performed, and graphic representations were produced using GraphPad Prism 8.2 (GraphPad Software; San Diego, CA, USA).

## 3. Results

**Figure 1:**
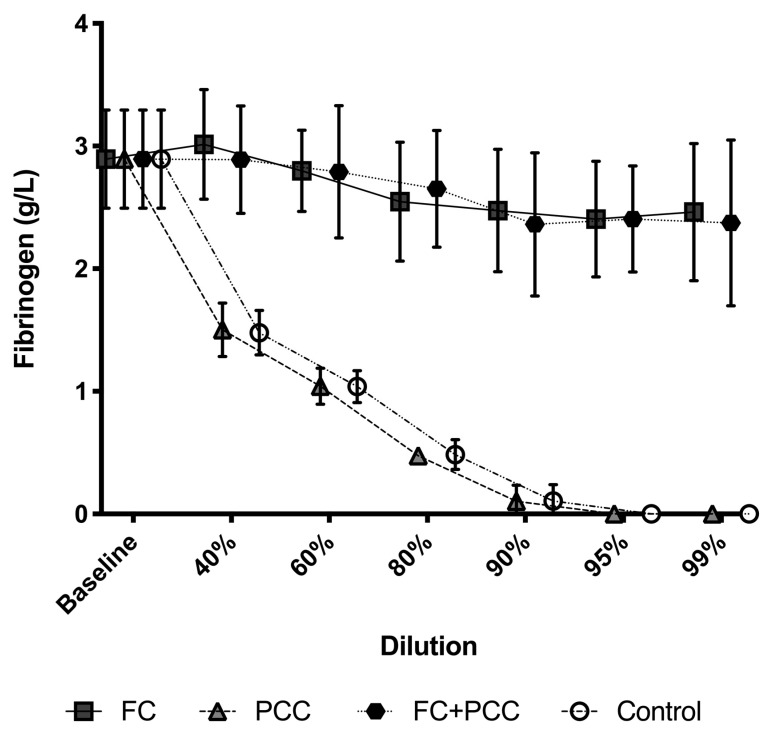
Fibrinogen concentrations in a comparison between the four study groups and different dilution levels. Significant differences (*p* < 0.001) occurred at all dilution levels between the groups containing fibrinogen concentrate (FC, FC+PCC) and those that did not (PCC, Control). FC, fibrinogen concentrate group; PCC, prothrombin complex concentrate group; FC+PCC, combined fibrinogen and prothrombin complex concentrate group; Control, control group.

Table 1 shows the baseline values of all measured parameters in undiluted platelet-free plasma. Fibrinogen concentrations were significantly higher at any dilution level in the groups containing fibrinogen concentrate (FC, FC+PCC) versus those not containing fibrinogen concentrate (Figure 1). No significant intra- or intergroup differences at distinct dilution levels occurred in the two groups containing fibrinogen concentrate (e.g., 99% dilution level: FC 2.5 (0.6) g/L in the FC group vs. 2.4 (0.7) g/L in the FC+PCC group; *p* > 0.999). In both the PCC group and the control group, fibrinogen concentrations decreased to 1.5 (0.2) g/L at the 40% dilution level and were unmeasurable at any dilution level above 90%.

**Figure 2:**
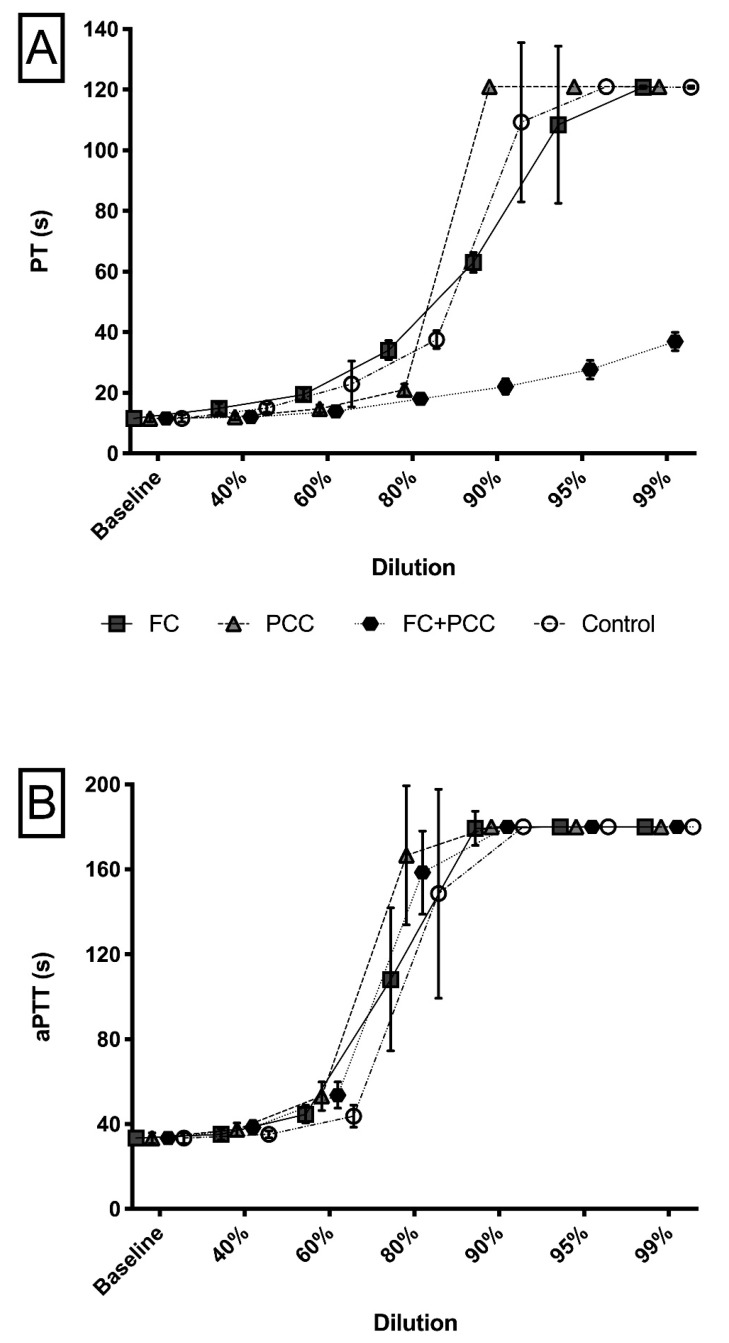
(**A**) Prothrombin time and (**B**) activated partial thromboplastin time in a comparison between the four study groups and different dilution levels. (**A**) Significant differences (*p* < 0.05) occurred at the 60% and 80% dilution levels between the groups containing PCC (PCC, FC+PCC) and those that did not (FC, Control), whereas, at the 95% and 99% dilution levels, only the FC+PCC group showed significantly different results in comparison to all other groups. (**B**) Significant intergroup differences (*p* < 0.05) occurred at the 80% dilution level only. PT, prothrombin time; aPTT, activated partial thromboplastin time; FC, fibrinogen concentrate group; PCC, prothrombin complex concentrate group; FC+PCC, combined fibrinogen and prothrombin complex concentrate group; Control, control group.

Figure 2 depicts the dilution-associated prolongation of the standard coagulation tests prothrombin time and activated partial thromboplastin time. With regard to prothrombin time, significant intergroup differences occurred at dilution levels greater than 60%, and only the FC+PCC group allowed for measurements throughout all dilution levels (Figure 2A). In all other groups, PT values were prolonged beyond the upper detectable limit of 120 s at varying dilution levels. In contrast, dilution-associated prolongation of the activated partial thromboplastin time showed a similar pattern in the comparison between the different study groups (Figure 2B). Dilution greater than 80% resulted in prolongation beyond the upper detectable limit of 180 s in all groups except for the FC group, with a mean activated partial thromboplastin time of 178 (12) seconds at the 90% dilution level.

Hemodilution led to a substantial progressive decrease in antithrombin activity, with no significant differences between the study groups at any dilution level (Figure S2, supplemental digital content). Factor II activity increased significantly in the two PCC groups (PCC, FC+PCC) in comparison to the baseline value (40% dilution level PCC 151% (16%) and FC+PCC 149% (11%); *p* = 0.0023 and 0.034, respectively). It was significantly higher at any dilution level in the two PCC groups, with a progressive decline to activities beyond 5% in the FC and control groups at the 99% dilution level (Figure S3, supplemental digital content).

**Figure 3:**
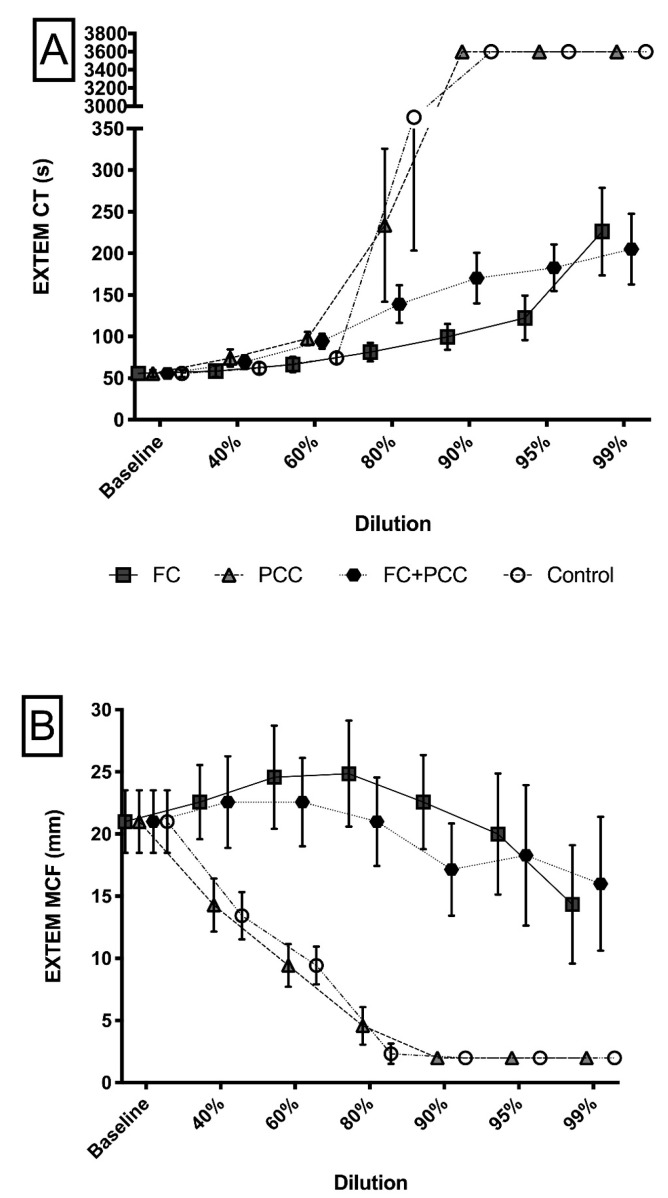
(**A**) EXTEM clotting time and (**B**) EXTEM maximum clot firmness in a comparison between the four study groups and different dilution levels. (**A**) Significant differences (*p* < 0.05) occurred at all dilution levels beyond 40%. Specifically, at the 60%, 80%, 90% and 95% dilution levels, the FC group showed significantly lower values (*p* < 0.05) in comparison with the FC+PCC group. (**B**) At any dilution level, groups containing fibrinogen concentrate (FC, FC+PCC) showed significantly higher (*p* < 0.001) values in comparison with those that did not (PCC, Control). The FC group showed significantly higher values (*p* < 0.05) in comparison with the FC+PCC group at the 60%, 80%, and 90% dilution levels. EXTEM CT, EXTEM clotting time; EXTEM MCF, EXTEM maximum clot firmness; FC, fibrinogen concentrate group; PCC, prothrombin complex concentrate group; FC+PCC, combined fibrinogen and prothrombin complex concentrate group; Control, control group.

ROTEM measurements yielded significantly different results in a comparison between the study groups. In the PCC and the control group, ROTEM measurements showed a flat-line pattern at all dilution levels beyond 80%, i.e., EXTEM CT values yielded results beyond the upper detectable limit of 3600 s and EXTEM MCF values were below 2 mm (Figure 3). This stood in sharp contrast to the FC and FC+PCC groups, where EXTEM CT and EXTEM MCF were measurable at all dilution levels. Although EXTEM MCF values were lowest at the 99% dilution level in the FC group and the FC+PCC group (14.3 (4.8) and 16.0 (5.4) mm, respectively), these differences did not reach statistical significance in comparison with the baseline values (*p* = 0.176 and 0.555, respectively). Remarkably, there was a significant difference in EXTEM CT values between the FC group and the FC+PCC group, with more pronounced dilution-associated prolongations of the EXTEM CT in the FC+PCC group up to a dilution level of 95% (Figure 3A). Likewise, EXTEM MCF values were significantly reduced in the FC+PCC group up to a dilution level of 90% when compared with the FC group (Figure 3B).

**Figure 4:**
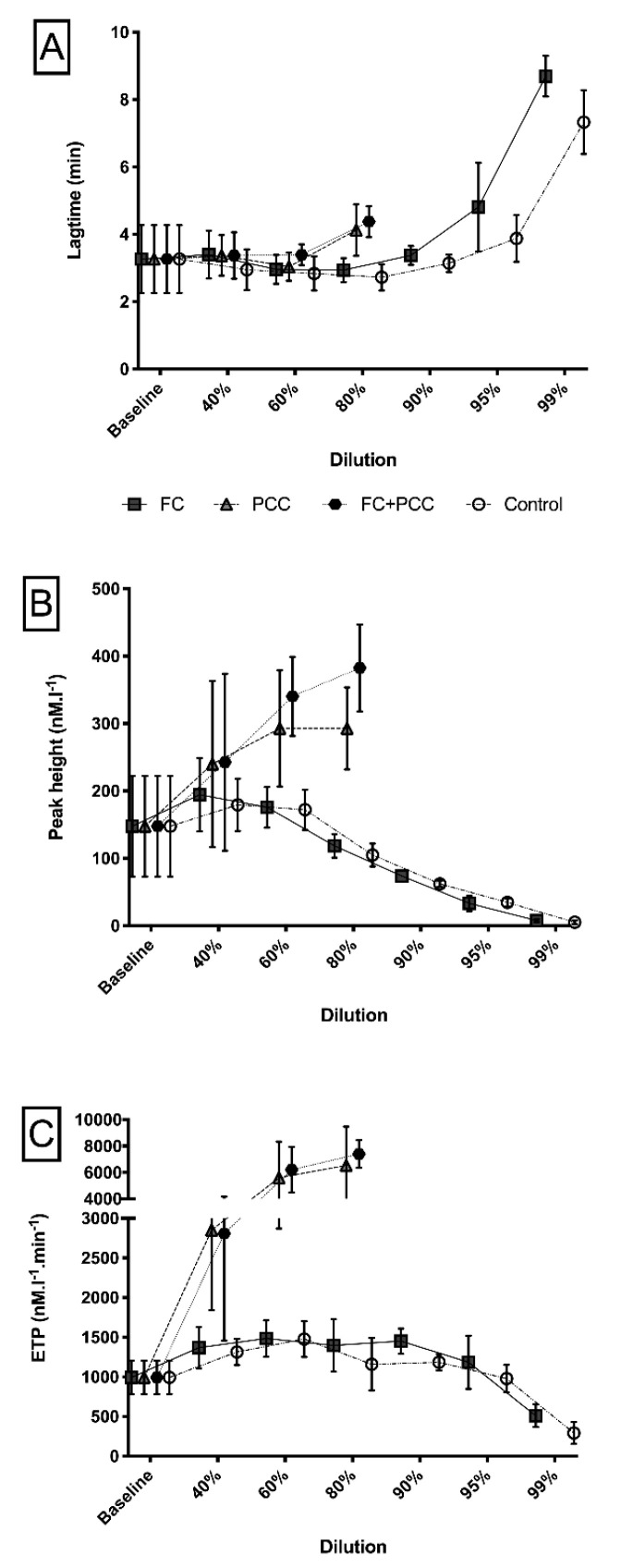
(**A**) Lagtime, (**B**) peak height, and (**C**) endogenous thrombin potential in a comparison between the four study groups and different dilution levels. (**A**) Significant differences (*p* < 0.05) between the groups containing PCC (PCC, FC+PCC) and those that did not (FC, Control) occurred at the 80% dilution level. (**B**) Within the groups containing PCC, FC + PCC showed significantly higher values (*p* < 0.05 at the 80% dilution level. (**C**) Groups containing PCC (PCC, FC+PCC) showed significantly higher values (*p* < 0.001) at the 40%, 60%, and 80% dilution levels, whereas no significant differences occurred between the two groups containing PCC. ETP, endogenous thrombin potential; FC, fibrinogen concentrate group; PCC, prothrombin complex concentrate group; FC+PCC, combined fibrinogen and prothrombin complex concentrate group; Control, control group.

With regard to thrombin generation measurements, Figure 4 illustrates differences in lag time, peak height, and endogenous thrombin potential between the study groups. At any dilution level higher than 80%, thrombin generation was not measurable in both PCC groups (PCC, FC+PCC). In the FC group and the control group, however, measurements of lagtime remained at roughly similar values up to a dilution level of 90% (Figure 4A). In contrast, peak height showed increasing values up to a dilution level of 60%, with a decreasing pattern at higher dilution levels only in these two groups (Figure 4B). Figure 4C illustrates measurements of endogenous thrombin potential that yielded results at or above the baseline values up to a dilution level of 95% for the FC group and the control group. In comparison with the baseline value, endogenous thrombin potential exhibited significantly higher results at the 60% dilution level in both groups (FC 667 (231) nM⋅L^−1^⋅min^−1^, *p* = 0.0272; control 643 (226) nM⋅L^−1^⋅min^−1^, *p* = 0.0312). At the 99% dilution level, endogenous thrombin potential was significantly lower (FC 509 (143) nM⋅L^−1^⋅min^−1^ and control 293 (139) nM⋅L^−1^⋅min^−1^) in comparison with the baseline value (*p* = 0.0001 and 0.0315, respectively).

**Figure 5:**
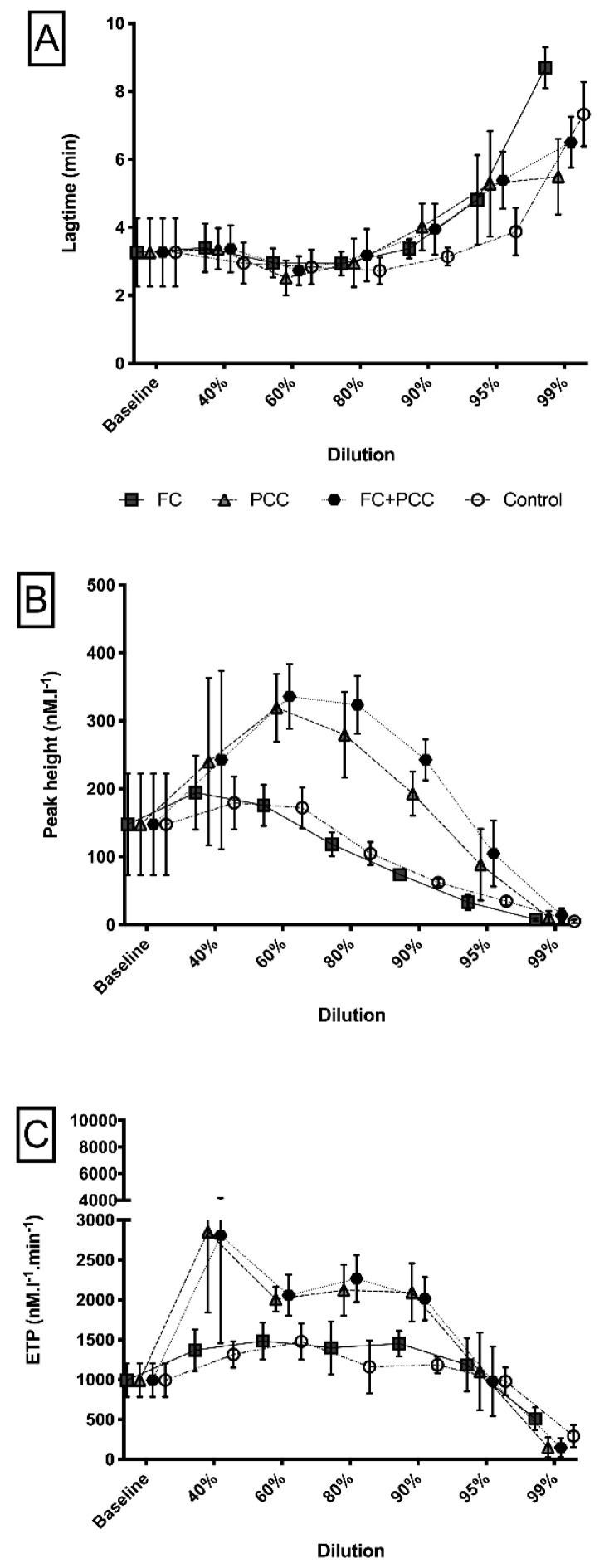
(**A**) Lagtime, (**B**) peak height, and (**C**) endogenous thrombin potential in a comparison between the four study groups and different dilution levels after correction of antithrombin activity to 100%. (**A**) The control group showed significantly shorter time values (*p* < 0.05) at the 90% and 95% dilution levels. (**B**) Within the groups containing PCC, FC+PCC showed significantly higher values (*p* < 0.05) at the 80% and 90% dilution levels. The groups without PCC (FC, Control) showed significantly lower values at all dilution levels, except for the 99% dilution level. (**C**) Groups containing PCC (PCC, FC+PCC) showed significantly higher values (*p* < 0.001) at the 40%, 60%, 80%, and 90% dilution levels, whereas no significant differences occurred between the two groups containing PCC. ETP, endogenous thrombin potential; FC, fibrinogen concentrate group; PCC, prothrombin complex concentrate group; FC+PCC, combined fibrinogen and prothrombin complex concentrate group; Control, control group.

When repeating thrombin generation measurements after substitution of antithrombin to a target of 100% activity in the two PCC groups (PCC, FC+PCC), lagtime showed a similar pattern between the groups with dilution-associated prolongations throughout all study groups (Figure 5A). However, measurements of peak height and endogenous thrombin potential suggest significantly increased thrombin generation up to a dilution level of 90% despite the correction of antithrombin activity in the PCC group and the FC+PCC group (Figure 5B,C).

## 4. Discussion

To the best of our knowledge, this is the first study to report the influence of four-factor prothrombin complex concentrate plus fibrinogen concentrate on hemostasis in a model of severe hemodilution, up to 99%. The current study revealed that up to a dilution level of 95%, viscoelastically determined clotting time was shortest in the study group substituted with fibrinogen only. Moreover, both endogenous thrombin potential and viscoelastically determined clot firmness remained at relatively stable values up to a dilution level of 95% with the substitution of fibrinogen but not prothrombin complex concentrate. In contrast, the substitution of prothrombin complex concentrate with or without fibrinogen concentrate led to excessive thrombin generation. Despite the normalization of antithrombin activity, endogenous thrombin generation exceeded baseline values after prothrombin complex concentrate substitution up to dilution levels of 90%.

Fibrinogen has been shown to be the first coagulation factor to reach critically low levels (i.e., <1.5–2 g/L) in bleeding patients [7]. In the present study, diluting plasma by 40% already reduced fibrinogen concentrations to values of 1.5 (0.2) g/L in both the PCC group and the control group, whereas fibrinogen concentrations remained relatively stable in the FC group and the FC+PCC group. These findings were mirrored by viscoelastic measurements of maximum clot firmness (MCF). In both fibrinogen-substituted groups (FC and FC+PCC), EXTEM MCF did not show statistically significant reductions of fibrinogen up to dilution levels of 99%, whereas in the PCC group and the control group, MCF values showed a dilution-associated decreasing pattern comparable to that of fibrinogen concentrations.

Being one of the clinically pertinent findings of our study, EXTEM MCF values confirmed that stable fibrinogen levels in the FC group did indeed allow for clot formation up to dilutions of 99% without adding further factor concentrates. This is in line with earlier studies showing similar results, albeit up to lower dilution levels only [20,21,22], and underlines the clinical importance of early fibrinogen substitution in bleeding patients, as recommended by current guidelines [6,23]. Furthermore, with regard to factor concentrates, our findings suggest that even in severely bleeding patients, the substitution of fibrinogen might be entirely sufficient to maintain normal levels of hemostasis, given the patients are not anticoagulated. Our results are in line with a recently published trial, reporting the use of fibrinogen concentrate only in patients with extreme blood loss of up to 11.8 L during thoraco–abdominal aortic aneurysm surgery [24].

Thrombin generation measurements further corroborated these findings, with endogenous thrombin potential values remaining at relatively stable values throughout a broad range of dilution levels of up to 95% without substitution of prothrombin complex concentrate. We observed an increase of thrombin generation potential at the 60% dilution level that has been described before [25,26] and is most likely attributable to a dilution-associated imbalance of endogenous pro- and anticoagulatory proteins [27,28]. The fact that endogenous thrombin potential did not decrease significantly without substitution of any coagulation factors in the control group, up to dilution levels of 95%, suggests that even in severely bleeding trauma patients, thrombin generation might be sufficient to achieve adequate hemostasis. This hypothesis is consistent with a recently reported study that found preserved thrombin generation in an animal model of severe trauma bleeding [29].

Furthermore, trauma patients exhibit increased thrombin generation in comparison with healthy controls [26,30,31,32]. Importantly, patients hospitalized after trauma encounter a substantially elevated risk of venous thromboembolism, and elevated thrombin generation has been shown to be an independent predictor for venous thromboembolic events in this patient cohort [33,34]. The concern for an elevated thromboembolic risk associated with excessive thrombin generation following administration of prothrombin complex concentrate has been expressed before [28,35,36]. Based on the results of phase III trials, administration of prothrombin complex concentrate is well established and recommended as first-line therapy for the reversal of vitamin K antagonists [37]. Similarly, several studies imply that prothrombin complex concentrate might also be effective for the reversal of non-vitamin K oral anticoagulants in bleeding patients [38]. However, prothrombin complex concentrate is increasingly used in an attempt to correct trauma-induced coagulopathy in patients outside these indications [12,14,28,39]. Our findings of an excessive overshoot of thrombin generation in the two groups substituted with prothrombin complex concentrate (PCC, FC+PCC) further highlight concerns over an increased thromboembolic risk ensuing the use of prothrombin complex concentrate in bleeding patients, which might be aggravated by an imbalance of pro- and anticoagulatory proteins. Recent data from an experimental animal study have shown that high-dose prothrombin complex concentrate monotherapy for trauma-related bleeding can increase hypercoagulation and aggravate the risk of disseminated intravascular coagulation [36,40]. Additional treatment with antithrombin was effective in mitigating prothrombin complex concentrate-induced hypercoagulation, supporting future investigation of this treatment approach in human patients. To guide the effect of prothrombin complex concentrate, individual assessment of thrombin generation potential should be undertaken. However, due to technical constraints, turnaround time, and availability, thrombin generation measurement for the management of acutely bleeding patients is not feasible in the clinical setting to date.

Typically, viscoelastically guided protocolized treatment algorithms for bleeding patients first aim to restore fibrinogen levels, as measured by tests focusing on functional fibrinogen [14,39]. As shown by a recent study, the substitution of fibrinogen concentrate alone resulted in a reduction of prolonged EXTEM clotting time [41]. Therefore, administration of prothrombin complex concentrate should only be considered once adequate fibrinogen restoration has been confirmed by clot firmness in platelet-inhibited tests [14,39]. Remarkably, we observed prolonged EXTEM CT values, together with decreased EXTEM MCF values, in the FC+PCC group in comparison with the FC group throughout a broad range of dilution levels. This rather surprising finding has already been reported recently in a different study conducted by our group [35]. We hypothesized that the heparin content of the prothrombin complex concentrate might play a role, as suggested by an earlier study [42]. However, the manufacturer of the reagent used for this study claims that EXTEM measurements should be insensitive to heparin contents of up to 5 IU/mL, and the results of a study examining different preparations of prothrombin complex concentrate in diluted blood do not support this hypothesis [43]. Therefore, the clinical relevance of this finding and its impact on currently propagated protocolized treatment algorithms for bleeding patients remain to be evaluated by further research.

With regard to conventional coagulation parameters, our study further underlines the major drawbacks of these tests for guiding hemostatic therapy in bleeding patients. Not only do they have relevantly prolonged turnaround times in comparison with point-of-care-available viscoelastic tests [14], but they also fail to adequately reflect a clinically relevant picture of hemostasis [44]. It has been known for a long time that prothrombin time and activated partial thromboplastin time measurements stop at a time point when only minimal amounts of thrombin have been generated [45]. This is consistent with the findings of our study. For example, values of activated partial thromboplastin time were significantly prolonged at the 80% dilution level in both PCC groups (PCC, FC+PCC), whereas endogenous thrombin potential measurements yielded results approximately 6-fold higher than baseline values.

Relevant limitations must be borne in mind when interpreting the results of our present study. First, due to the experimental in-vitro setup, excluding cell-based elements of the coagulation process and the small sample size of our pilot study, our results cannot readily be transferred into clinical practice. Future studies, including cell-based aspects of coagulation such as the endothelium, platelets, or erythrocytes, might help put our findings into context with in-vivo coagulation processes. Furthermore, our findings should prompt prospective randomized clinical trials to further investigate the relevance of thrombin generation measurements in different clinical settings as well as the safety and efficacy of using prothrombin complex concentrate in an attempt to correct thrombin generation. Second, we did not investigate different types of preparations of prothrombin complex concentrate (e.g., heparin-free preparations) that might have led to distinct results due to their different compositions [43]. Third, in particular, the higher levels of dilution described in the present study do not represent hemodilution levels encountered in clinical practice. Finally, a number of different viscoelastic devices are used for the management of bleeding patients and our findings might not be readily translated to measurements obtained by other viscoelastic devices. Especially with regard to the observed EXTEM CT prolongations in the FC+PCC group versus the FC group, this calls for further research to better define the role of prothrombin complex concentrate in protocolized hemostatic management algorithms for bleeding trauma patients.

## 5. Conclusions

In an in-vitro hemodilution model, throughout a broad range of dilution levels of up to 95%, the substitution of prothrombin complex concentrate was not necessary to provide for both viscoelastically determined clot formation as well as sufficient thrombin generation. In contrast, the substitution of prothrombin complex concentrate resulted in an overshoot of thrombin generation that might be particularly worrisome in trauma patients with an increased risk of thromboembolism once bleeding has stopped. Fibrinogen substitution, however, played a key role in the preservation of hemostatic capacity. The results of our present study scrutinize currently propagated hemostatic treatment algorithms and call for further research regarding the role of prothrombin complex concentrate in bleeding patients.

## Figures and Tables

**Table 1:** Baseline values of plasma coagulation tests, thromboelastometry (ROTEM) parameters, and thrombin generation measurements obtained from platelet-free plasma. Values are mean (SD).

Plasma coagulation tests	Fibrinogen; g/L	2.9 (0.4)
	Prothrombin time; seconds	11.9 (2.1)
	Activated partial thromboplastin time; seconds	34.9 (3.5)
	Factor II activity; %	106 (4)
	Antithrombin activity; %	107 (5)
ROTEM parameters	EXTEM CT; seconds	55 (4)
	EXTEM MCF; mm	21 (3)
Thrombin generation	Lag time; min	3.3 (1.0)
	Peak height; nM·L^−1^	148 (75)
	Endogenous thrombin potential; nM·L^−1^⋅min^−1^	993 (210)

## Supplementary Materials

The following are available online at https://www.mdpi.com/2077-0383/9/12/3805/s1. Figure S1: Schematic description of the experimental procedures.; Figure S2: Antithrombin activity in a comparison between the four study groups and different dilution levels; Figure S3: Factor II activity in a comparison between the four study groups and different dilution levels.
