# Supplementary material for: Sufficient Thrombin Generation Despite 95% Hemodilution: An In Vitro Experimental Study
Source: J Clin Med. 2020 Nov 25;9(12):3805. doi: 10.3390/jcm9123805 (PMC7760770; doi:10.3390/jcm9123805)
Supplement: Supplementary file 1 [file jcm-09-03805-s001.pdf]

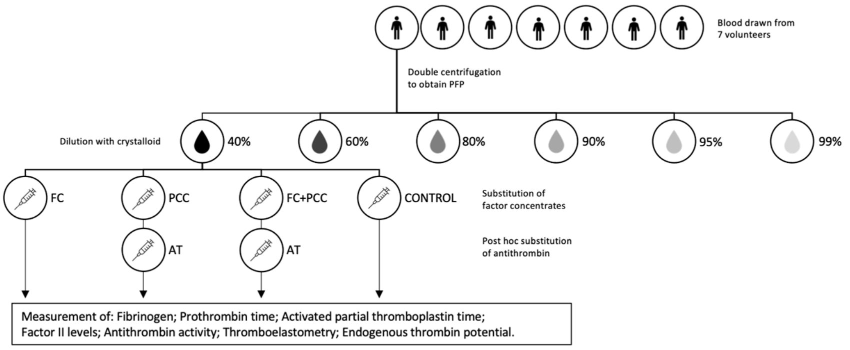

Figure S1. Schematic description of the experimental procedures. AT, antithrombin; FC, fibrinogen concentrate; FC + PCC, combined fibrinogen and prothrombin complex concentrate group; PCC, prothrombin complex concentrate group; PFP, platelet free plasma; Control, control group.

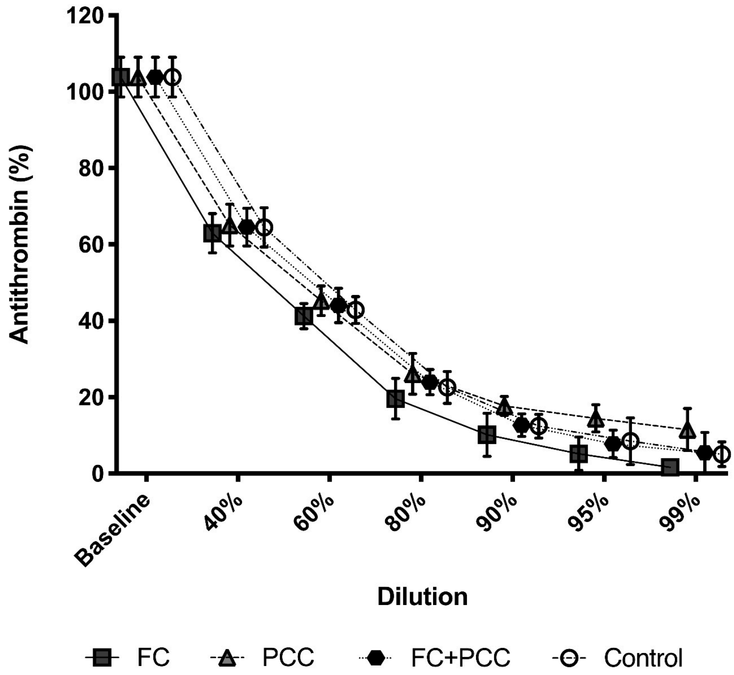

Figure S2. Antithrombin activity in a comparison between the four study groups and different dilution levels. No significant intergroup differences occurred at any dilution level. FC, fibrinogen concentrate group; PCC, prothrombin complex concentrate group; FC + PCC, combined fibrinogen and prothrombin complex concentrate group; Control, control group.

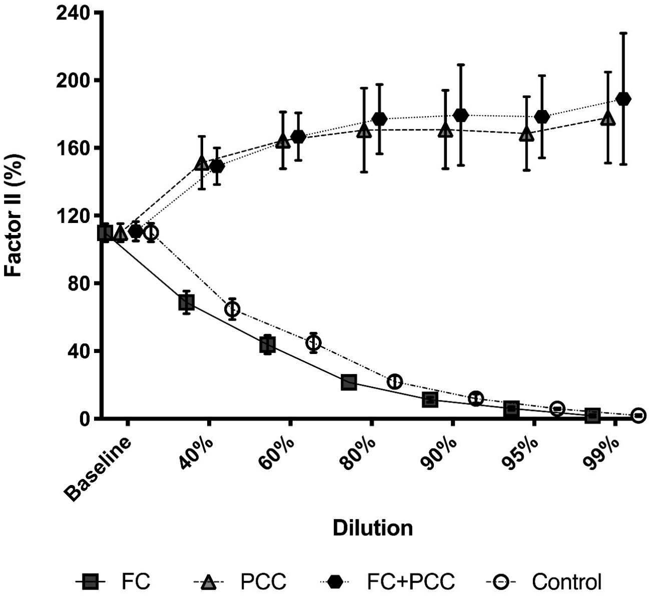

Figure S3. Factor II activity in a comparison between the four study groups and different dilution levels. Significant differences ($p < 0.05$) occurred at all dilution levels between the groups containing PC (PCC, FC+PCC) and the groups that did not (FC, Control). FC, fibrinogen concentrate group; PCC, prothrombin complex concentrate group; FC + PCC, combined fibrinogen and prothrombin complex concentrate group; Control, control group.
